# Toward a new personalized psycho-social approach for the support of prostate cancer and their caregivers dyads: a pilot study

**DOI:** 10.3389/fmed.2024.1356385

**Published:** 2024-04-04

**Authors:** Clizia Cincidda, Silvia Francesca Maria Pizzoli, Serena Oliveri, Paolo Guiddi, Gabriella Pravettoni

**Affiliations:** ^1^Applied Research Division for Cognitive and Psychological Science, IEO European Institute of Oncology IRCCS, Milan, Italy; ^2^Department of Oncology and Hemato-Oncology, University of Milan, Milan, Italy; ^3^Facoltà di Psicologia, Università Cattolica del Sacro Cuore, Milan, Italy; ^4^“Aldo Ravelli” Center for Neurotechnology and Brain Therapeutics, Department of Health Science, DISS, University of Milan, Milan, Italy; ^5^Neurological Clinic, ASST-Santi Paolo e Carlo, Milan, Italy

**Keywords:** prostate cancer patients, dyads, caregivers, chronic disease, decision making

## Abstract

**Introduction:**

Prostate cancer patients (PCP) often struggle with a significant emotional, physical, and social burden during the care-flow pathway. Noteworthy, PCP should not be considered a standalone patient, but someone who is connected with a relevant social environment and that is usually supported by a beloved one, the caregiver. The involvement of the caregivers through the care pathway might bring significant benefits both on the psychological and the treatment and decision-making side. The present pilot study aimed at preliminarily assessing quantitatively the psychological impact of a prostate cancer diagnosis on the degree of agreement of PCPs and their caregivers on medical decisions, coping resources and psychological distress levels.

**Methods:**

16 PCP and their caregivers were enrolled in the study and fulfilled a battery of standardized questionnaires.

**Results:**

Results showed low concordance in decision making styles and preferences in patients and their caregivers and that the dyads showed similar depression symptoms levels. Relevant features of the psychological needs of the analyzed dyads, such as need for information and support, also emerged.

**Conclusion:**

On the basis of these preliminary results, guidelines for the construction of tailored brief psychological support interventions for PCP dyads are provided.

## Introduction

In 2020, it was estimated that prostate cancer (PC) accounts for 7% of all cancers, although survival rates were very high (1, 2). Indeed, with early PC diagnosis, patients could have more favorable survival outcomes, leading PC to be treated as a chronic disease (3). However, screening for prostate cancer remains a highly debated topic in both clinical and public health sphere due to the unnecessary diagnosis and treatment of otherwise slow-progressing cases. Even today, the test for elevated levels of prostate-specific antigen (PSA) in blood com screening tool is still used, although we know its limitations in use, as it often gives false positives (4). To date, there are several treatments for localized PC, that can be divided into active treatment (e.g., radical prostatectomy, external beam radiation therapy), and active surveillance (AS) (2, 3, 5). The active treatments may have significant and potential side effects affecting urinary, sexual, hormonal, and bowel function (e.g., erectile dysfunction, reduced libido). These aspects can have further negative psychological implications, causing anxiety, depression, fatigue, stress, pain, and FCR (2, 3, 6–9). Moreover, the sexual challenges, that PC patients may face, can impact patient’s intimate relationships and lead to feelings of frustration and loss of self-confidence (1, 10). On the other hand, AS allows patients with low-risk PC to avoid active treatment and thus the associated adverse effects (2, 11). However, patients have to undergo PSA testing and digital rectal examinations at regular intervals and annual/biannual biopsies (2, 11). Thus, even patients under the AS reported high level of anxiety and depression, although they did not have the side effect of an active treatment (11).

In recent years, the decision between active treatment or active surveillance is shared with the patient. Indeed, there has been a shift from a paternalistic approach, in which physicians made decisions without considering the patient’s opinion, to a patient-centered approach, in which the patient is an active participant of care (12–14). In recent years there is a greater focus on patients’ individual preferences, needs, and values, as well as considering the clinical aspects of the disease, when discussing DM (15, 16). Consequently, treatment decisions should be made collaboratively between patients and physicians, with a two-way exchange of information (17–19). The shared decision-making seems to be the conceptual approach to decision making that best fits the patient-centered approach, in which patients and physicians have different but equally valuable perspectives and roles (20). However, the complexity of available treatment options and the potential consequences of these decisions can lead to decision-making anxiety; thus, it would be appropriate to provide precise information on the possible effect of the treatment and, if necessary, to involve a caregiver in the decision-making process (DM) (2, 21).

Adopting a biopsychosocial standpoint, patients are characterized within the context of their relationships and rely on their “significant others” to guide DM, that means not only family caregivers, but also physicians (6, 20, 22, 23). Recently, authors such as Rapley (24), who introduced the concept of “distributed DM” or Epstein and Street (25), who introduced the concept of “share mind,” have pointed out how decisions can be made in the context of social interactions that in turn can influence them (20). Based on this, some authors have introduced collaborative decision-making models including not only the physician and patient, but also family caregivers, such as Elwyn’s collaborative deliberation model (26) or Légaré et al.’ (27) interprofessional shared DM model (IP-SDM) (20). However, it is only recently that the roles and dynamics of family caregivers within the DM process have been delineated through the development of the so-called TRIO-framework. According to this theoretical framework, the decision-making process could be represented graphically by a triangle, as it succeeds in capturing and expressing the complex extent of physician-patient-family caregiver influence on a decision. Although the clinician plays a dual role within triadic DM, i.e., as a participant and facilitator, having medical expertise and a professional role in the DM process, this model emphasizes “equal” triadic sharing of a decision among the three actors: patient, physician, and caregiver. The family caregivers’ involvement in DM can vary from passive to active, depending on the illness trajectory or severity, personal characteristics (e.g., demographic, psychological, relational, cultural, and medical) and type of relationship among patients and caregivers and among the extend family (20).

In general, along the cancer journey, patients and caregivers explore together treatment options, weigh risks and benefits, and consider the impact on quality of life (28). The presence of a caregiver may help patients to better cope with the cancer diagnosis, its subsequent treatments, and the treatment decision-making (29–31). Quite often, patients are accompanied by their caregivers during the visits to be supported in the interaction and communication with the oncologists (14, 32). Participating in medical visits, caregivers can state their opinions, preferences, and beliefs on the treatment decisions (20, 33–36). Moreover, caregivers may feel entitled to influence patients’ decisions; they also inevitably bring a series of emotional reactions, interpersonal dynamics, and expectations (36–39). For both patients and caregivers, it is necessary to receive clear information that enables them to understand the diagnosis, treatment options, self-care and support available, in order to have a more active role in the DM (40, 41). A recent systematic review reported that patients and caregivers seemed to have similar views on their involvement in DM: most patients and caregivers dyads preferred to share the responsibility of the decision or that patients decide after seeking input from the caregivers (31, 42). Some researchers have used concordance/discordance of cancer communication as a measure of the level of agreement within dyads on the topics of cancer communication and decision making, showing that concordance/discordance in cancer communication is not static, but fluctuates during cancer treatment (43). However, it is not yet clear whether patients and caregivers agree on their involvement in decision making and whether a difference in involvement might depend on the type of relationship. Finally, caregivers’ involvement in the oncological examinations was associated with increased patients’ satisfaction with care, understanding of cancer-related information, treatment adherence, physical and mental health (38, 44, 45). In contrast, as a downside, higher caregivers’ involvement in DM was also associated with higher caregiver burden and psychological distress (46).

In conclusion, PC diagnoses may affect dyadic relationships, and in particular intimacy, dyadic communication about feelings, family management, and personal expectations about life (6, 10, 31). Recent research showed a reciprocal psychological influence between patients and caregivers after PC diagnosis, reporting that highly distressed patients have highly distressed caregivers (10, 32, 47). In such circumstances of crisis, the dyads co-create and share coping strategies to respond to the stressful event (29, 48). Patient and caregiver individual coping styles may have a mutual positive or negative impact on QoL and psychological status (29, 30). The common coping strategies used by dyads in this context are shared information seeking and SDM, exchanging worries, and efforts to manage their emotional reactions (20, 30, 31).

Based on these premises, this pilot study is aimed at assessing dyads share expectations about mutual involvement in cancer related decision making, their agreement and their mutual influence on the psychological adjustment. In particular, we expect a similar view between patient and caregiver regarding their involvement in DM, as reported in the recent systematic review (31). We formulated three hypotheses: (H1) high agreement among patients and caregivers regarding their involvement in DM and a good triadic involvement in DM; (H2) a good communication style within the dyads that may represent a good relationship and a protective factor for psychological distress; (H3) a similar impact of the cancer diagnosis on the dyads in terms of psychological distress.

## Materials and methods

### Participants

16 prostate cancer patients and their caregivers were invited and agreed to participate in this pilot study. The recruitment took place at the European Institute of Oncology of Milan (Italy). Patients accompanied by family caregivers during their oncology visits were flagged up by oncologists as possible study participants. The lead research psychologist contacted them by telephone to explain the purpose and procedures of the study.

Inclusion criteria for patients were as follows: (1) age 18 years or older, (2) recent diagnosis of prostate cancer (enrolled patients received the diagnosis in the two weeks prior to enrollment), (3) not currently decide the treatment to be undergone (when patients were enrolled, further diagnostic investigations were still being conducted in order to be able to determine which treatment was most appropriate for the diagnosis received), and (4) in sufficient physical and mental health to understand and complete the study. Patients who were diagnosed with early mental disorders (before age 40) or severe neurological disorder or advanced cancer stage (e.g., palliative patients) were excluded from the study.

After patients agreed to participate in the study, adult family caregivers (age ≥ 18) were also asked to participate in the study. Caregivers with an early mental disorder (before age 40) or severe neurological disorder were excluded from the study.

The study was conducted in accordance with the Declaration of Helsinki and approved by the Institutional Review Board (or Ethics Committee) of the European Institute of Oncology, IRCCS (n. R1598/21-IEO1702). Written informed consents were obtained.

### Procedure

The pilot study was conducted in November 2022. Patient/caregiver dyads, who agreed to participate in the study, completed a series of questionnaires separately that were administered electronically through the Qualtrics^™^ online platform. Basic medical data such as cancer diagnosis, stage, duration since diagnosis, and treatments administered were obtained from the hospital information systems of the participating facilities.

### Measures

The set of questionnaires completed by patients and caregivers consisted of the following. First, patients and caregivers were asked about sociodemographic characteristics such as age, gender, education level, occupation, origin, and type of family relationships. Then, a set of psychological questionnaires were administered:

As used in other studies (49–52), a modified version of the Control Preference Scale (CPS) questionnaire (53) assessed patients’ and caregivers’ decision-making control preference. Responses were rated as active (e.g., patients or caregivers preferred to be the decision maker), semi-active (e.g., patients or caregivers preferred to be the decision-makers while considering the opinion/preferences of the caregivers or patients, respectively), collaborative (e.g., patients and caregivers preferred to share the responsibility of the decisions), or passive (e.g., patients or caregivers preferred not to be the decision-makers, letting the caregiver or the patient decide, respectively). The CPS was adapted and cross-culturally validated in Italian in a sample of people with multiple sclerosis, showing a moderate test–retest reliability (49).

Five items from the Personal Assessment of Intimacy in Relationships scale [PAIR (54)] evaluated the degree of intimacy that each actor of the dyad currently perceives in the sphere of communication (we referred to it as the dyadic communication) (55). The response rate was on a 5-point Likert scale (from completely describing my relationship to not describing it). The difference between the two actors scores revealed the intimacy between the dyad (54). An example of the item was “My partner listens to me when I need someone to talk to.” The questionnaire was not validated in Italian, so we used the back translation method to create the Italian version. The internal reliability coefficient (Cronbach α) of the communication subscale of PAIR was 0.80 (55), in our study was 0.83, confirming the high internal reliability.

The Miller Behavioral Style Scale [MBSS (56)] aimed to determine the information-seeking behavior (a coping style) of threatened individuals and classified them as active information seekers or stressful situation avoiders. Four fictional stress-inducing situations (i.e., a dentist, hostage, redundancy, and airplane scenario) were presented and participants were asked to select one or more of the eight statements representing the monitoring coping style (e.g., paying attention, scanning, and amplifying potentially painful or harmful aspects of information and experiences related to illness, such as “I would watch all the dentist’s movements and listen for the sound of the drill”) or the blunting coping style (e.g., avoiding, distracting from medical information, such as “I would do mental puzzles in my mind”). This psychological variable can be considered a trait variable that remains stable over time. Cronbach’s α coefficients for the monitoring and blunting sub-scales were 0.65 and 0.41, respectively (57), and in our study were 0.743 and 0.516, respectively. However, this scale was not validated in Italian, so we used the back translation method to create the Italian version.

The Hospital Anxiety and Depression Scale [HADS (58)] consisted of 14 items on a person’s mood in the past week (seven items assess depression, such as “I still enjoy the things I used to enjoy” and seven items assess anxiety, such as “I feel tense or wound up”) (59). Each item is rated on a 4-point scale (from not at all to most of the time) for a total score ranging from 0 to 21 for each subscale. A higher score indicates higher distress and the cut-off points for establishing the presence of anxiety and depression is set at 8. This scale has been adapted and validated into Italian both for cancer patients and a community sample (60, 61). Cronbach’s alpha for anxiety varied from 0.68 to 0.93 (mean 0.83) and for depression from 0.67 to 0.90 (mean 0.82) (62, 63).

The Multidimensional Scale of Perceived Social Support [MSPSS (64)] was composed by 12 items rated on a seven-point Likert scale (from strongly disagree to strongly agree). Participants were asked to indicate how they feel about each statement (e.g., “I have a special person who is a real source of comfort to me”). Scoring can be calculated in terms of a total score by summing the scores of all 12 items. The sample can be divided into groups based on the total score (12–35 = low support; 36–60 = moderate support; 61–84 = high support). The scale has been adapted and validated in Italian by Di Fabio and Palazzeschi (65). In our study, the internal reliability coefficient (Cronbach α) was 0.94.

The SF-12 Health Survey is composed of 12 items, selected from the SF-36, evaluating the day they completed the questionnaire and the previous 4 weeks. The scoring provides two summary measures related to physical and mental aspect of health (PCS-12 and MCS-12). Scores range from 0 to 100, with higher scores indicating better physical and mental health functioning. According to Ware and colleagues (66), the SF-12 Physical and Mental Summary Scales could be scored as follow: a score of 50 or less on the PCS-12 has been recommended as a cut-off to determine a physical condition, while a score of 42 or less on the MCS-12 may be indicative of ‘clinical depression’. Kodraliu et al. (67) assessed the SF-12 in various Italian settings, including the general population and specific patient groups, showing that the SF-12 has good validity. The mean scores reported by the authors for the general population were 47 (SD 9.61) and 46.2 (SD 10.51) for PCS and 46.5 (SD 10.6) and 44.8 (SD11.4) for MCS. Regarding the out-patients, the mean scores were 43 (SD = 5.2) and 40.4 (SD = 9.7) for PCS and 44.1 (SD = 6.3) and 44.0 (SD = 11.2) for MCS.

The 9-item Shared Decision-Making Questionnaire (SDM-Q-9) consisted of an open-ended question designed to explore the decision-making context as well as 9 multiple-choice questions rated on a six-point Likert scale, from completely disagree to completely agree (68). An example item was “My doctor wanted to know exactly how I want to be involved in making the decision.” The total raw score was obtained by summing all items ranging from 0 to 45. If there were one or two items missing, the average of the completed items could be used to calculate the raw score. The authors suggested multiplying the raw score by 20/9 resulting in a transformed score ranging from 0 to 100, where 0 indicates the lowest possible level of SDM and 100 indicates the highest possible level of SDM. The SDM-Q-9 was translated into English and Italian, allowing for use in international research (68). The questionnaire was validated in a psychiatric clinical sample showing a Cronbach’s α coefficient of 0.86 (69). In our study, Cronbach’s α was 0.862, showing a high internal consistency.

The Consultation and Relational Empathy (CARE) Measure (70) evaluated doctor’s communication and relational empathy during the consultation (e.g., How was the doctor at fully understanding yours concerns?”). It consisted of 10 items rated on a six-point Likert scale, ranging from poor to excellent. Moreover, participants could select the option “does not apply.” Scores ranged from the lowest score (10) to the highest one (50), with a higher score meaning excellent empathy shown by doctors. Mercer and Murphy (71) assessed the CARE’s performance and suitability in secondary care showing that the mean score for the total sample of patients was 43.5 (variance 55.8, standard deviation 7.47, *N* = 1,010). The Italian version of the CARE measure showed high internal reliability (Cronbach’s α = 0.962) (72), that was confirmed in our study (Cronbach’s α = 0.97).

### Statistical analysis

Descriptive statistics were calculated on raw data to report participants’ socio-demographic characteristics (mean, standard deviation, median, minimum and maximum or reported frequencies in combination with confidence intervals). Dyadic analyses were conducted to verify the differences between patients and caregivers and their interdependence for each variable of interest. Specifically, t-tests, contingency tables and Chi-Square tests were then performed to compare patients and caregivers. Expected values and residuals in every box were calculated to verify if a specific group gave a significantly higher or lower rate of response (observed values) to certain items, compared to the percentage expected and calculated on the number of subjects recruited. Finally, correlations analyses were conducted for the sample of patients, caregivers and dyads. Analyses were performed with SPSS (25.0, IBM, United States, 2014).

## Results

### Descriptive analysis of the sample

16 dyads were enrolled in the study composed by 16 prostate cancer patients (all male, Mage = 66.13, SD = 7.402) and their caregivers (12 females and 4 males, Mage = 57.06, SD = 10.853). 13 dyads had a marriage/partner relationship (81.3%), and 3 had a kin relationship (18.8%). Socio-demographic data are reported in Table 1.

**Table 1:** Descriptive statistics for patient and caregiver demographic variables.

Variable	Patient (*N* = 16)	Caregiver (*N* = 16)
Age (M ± SD, range)	66.13 ± 7.402 55–76	57.06 ± 10.853 35–74
*Gender*		
Male		25%
Female		75%
*Educational level*		
Primary/middle school	18.8%	18.8%
High school	50%	31.3%
Bachelor/Master’s Degree	31.2%	43.8%
Post PhD	0%	6.3%
*Employment*		
Blue-collar	31.3%	62.5%
White-collar	25%	12.6%
Unemployed	6.3%	6.3%
Retired	37.5%	18.8%
*Origin*		
North of Italy	43.75%	43.75%
Center of Italy	31.25%	31.25%
South of Italy	25%	25%
*Cancer stage*		
Stage I or Gleason <6	37.5%	
Stage II or Gleason = 7	62.5%	

The preferred involvement in the shared decision-making.

Half of the patients (*n* = 8, 50%) and slightly less than half of the caregiver (*n* = 7, 43.8%) preferred to share decision-making responsibility within the dyads. The other half of the patients preferred to have an active role in decision-making, although almost all of the patients preferred to make decisions after taking the caregiver’s opinion into account (*n* = 7, 43.8%) and only one patient preferred to decide alone (6.3%). Regarding caregivers, slightly less than half of them preferred to let the patient having an active role in the decision-making, although some of them (*n* = 6, 37.5%) preferred the patients considered their opinion. Only two caregivers preferred to have an active role in the decision-making, although considering the patients standpoint (12.5%), one was a son, and one was a wife. No sons preferred to share the responsibility of the decision with the patients, that in this case was the father.

Considering the concordance among responses, 7 dyads (43.75%) agreed on the preferred decision-making modality, specifically 4 dyads agreed in sharing decision-making responsibility (25.5%). For more detail, see Table 2. Contingency table and Chi-square test didn’t show a significant association between patients’ preferences in decision-making modality and caregivers’ preferences. Indeed, pairwise t-test showed a statistical significant different between patients and caregivers’ preferences (t_(15)_ = −3.033, *p* < 0.01).

**Table 2:** Contingency table among patients’ and caregivers’ preferences in their decision-making involvement.

		Caregivers’ preferred involvement								
		Patients decide by himself		Patients decide after considering caregivers’ opinion		Patients and caregivers share the responsibility of the decision		Caregivers decide after considering patients’ opinion		Total
		*n*	%	*n*	%	*n*	%	*n*	%	*N* %
Patients’ preferred involvement	Patients decide by himself	0	0.0%	1	6.3%	0	0.0%	0	0.0%	1 6.3%
	Patients decide after considering caregivers’ opinion	1	6.3%	3	18.8%	3	18.8%	0	0.0%	7 43.8%
	Patients and caregivers share the responsibility of the decision	0	0.0%	2	12.5%	4	50.0%	2	12.5%	8 50.0%
Total		1	6.3%	6	37.5%	7	43.8%	2	15.5%	16,100%

Based on patients’ and caregivers’ concordance on their preferences regarding the involvement in the shared decision-making, we divided the sample into those who agreed and those who disagreed (Dummy variable = Agree 1, disagree 0). However, no differences were found in all the patients’ and caregivers’ psychological variables between dyads who agreed and who disagreed.

### Patients and caregivers’ communication style

Referring to the communication style, patients and caregivers reported experiencing an open and flowing exchange of ideas between them, showing high scores in the communication scale (Mp = 4.43, SD = 0.51; Mcg = 4.11, SD = 0.84). Table 3 shows the difference between patients’ and caregivers’ scores. More than half of the dyad agreed on their communication style (*n* = 9, 56.25% had same or very similar score), while within the other dyads, patients reported a better communication style than the caregivers (*n* = 5, 31.25%) or vice versa (*n* = 2, 12.5%). Patients’ communication style was positively correlated with patients’ perceived social support (*r* = 0.51, *p* < 0.05).

**Table 3:** PAIR differences between patients and caregivers.

Differences	*N*	%
≤ − 0.5	2	12.5
0	9	56.25
≥ 0.5	5	31.25
Total	16	100,0

How do patients and caregivers cope with the prostate cancer diagnosis?

Most of the patients and the caregivers showed a monitoring coping style in responding to breast cancer diagnosis (*n* = 10, 62.5% of both). This mean that most of patients and caregivers tended to pay more attention to, scan for, and amplify threatening cues. Only one caregiver showed to have a blunting coping style, and no one of the patients (6.25%). The other didn’t show a preference in the two coping styles (6 patients and 5 caregivers on 16).

After receiving the breast cancer diagnosis, patients showed higher level of anxiety and depression than the caregiver (Anxiety: Mp = 12.94, SD = 2.74; Mcg = 11.75, SD = 2.02; Depression: Mp = 11.00, SD = 1.59; Mcg = 9.06, SD = 1.06), however a significant difference was found only in the level of depression between patients and caregivers (t(15) = 4.20, *p* < 0.001). Moreover, there was no significant correlation between patients and caregivers’ anxiety and depression. Patients’ level of anxiety was related to patients’ mental health (*r* = 0.737, *p* < 0.01).

Regarding the QoL, patients and caregivers reported a mean score in the physical and mental subscale above the cut off (PCS: Mp = 53.23, SD = 3.73; Mcg = 53.11, SD = 6.88, MCS: Mp = 46.37, SD = 12.96; Mcg = 49.07, SD = 9.65). Comparing patients and caregivers’ score, no significant difference was found. There was a positive correlation between patients’ mental health and their age (*r* = 0.60, *p* < 0.5). Regarding caregivers, a negative correlation between their mental health and their physical health (*r* = −0.54, *p* < 0.05) was found, while their mental health was positively related to their perceived involvement in the SDM (*r* = 0.70, *p* < 0.01).

Finally, both patients and caregivers reported high level of perceived social support (Mp = 64.69, SD = 15.17; Mcg = 70.38, SD = 9.21). Caregivers perceived social support was positively related to their age (*r* = 0.63, *p* < 0.01) and their mental health (*r* = 0.56, *p* < 0.05), while was negatively related to the perceived involvement in the SDM (*r* = 0.56, *p* < 0.05).

### Triadic relationship: patients, caregivers, and medical team

Regarding the relationship with medical team, patients and caregivers reported to be sufficiently involved in the decision-making process by medical team (Mp = 77.92, SD = 21.07; Mcg = 71.58, SD = 29.57) and a good medical team’ empathy and ability to communicate during the oncological consultation (Mp = 40.31, SD = 9.80; Mcg = 46.79, SD = 12.22), however, patients showed lower score than the referred sample. No significant difference was found between patients and caregivers.

### Correlations between patients and caregivers

We run a bivariate correlation between the psychological variables of patients and caregivers (Table 4). We found that caregivers’ communication style was positively related to patients’ levels of anxiety (*r* = 0.54, *p* < 0.05), it means that when the caregivers thought to have an open and flowing exchange of ideas with the patients, it enhanced patients’ level of anxiety.

Moreover, it was found that high level of caregivers’ anxiety was related to lower level of patients’ depression (*r* = −0.56, *p* < 0.05). Moreover, caregivers’ anxiety was positively correlated with patients’ high score in medical team’ empathy and communications (*r* = 0.59, *p* < 0.05). Caregivers’ mental health was associated to patients’ perception of medical team’ empathy and communications (*r* = 0.56, *p* < 0.05). Whereas caregivers’ perception of medical team’ empathy and communications were negatively related to patients’ perceived involvement in the SDM (*r* = −0.56, *p* < 0.05).

Finally, patients’ perceived involvement in SDM was positively related to caregivers’ one (*r* = 0.65, *p* < 0.05).

**Table 4:** Patients and caregivers’ correlations.

Variable	n	M	SD	1.	2.	3.	4.	5.	6.	7.	8.	9.	10.	11.	12.	13.	14.	15.	16.	17.	18.
1. Age_p_	16	66.13	7.40	-																	
2. Age_cg_	16	57.06	10.85	0.11	-																
3. PAIR_p_	16	4.43	0.51	0.25	0.12	-															
4. PAIR_cg_	16	4.11	0.84	0.15	0.12	0.06	-														
5. HADS-A_p_	16	12.94	2.74	0.30	−0.34	−0.11	0.54*	-													
6. HADS-A_cg_	16	11.75	2.02	−0.11	−0.45	0.47	−0.07	0.18	-												
7. HADS - D_p_	16	11	1.59	−0.23	0.29	−0.33	0.00	−0.40	−0.56*	-											
8. HADS - D_cg_	16	9.06	1.06	0.25	−0.01	0.05	0.23	0.02	−0.24	0.08	-										
9. MSPSS_p_	16	64.69	15.17	0.18	−0.12	0.51*	0.43	0.37	0.21	−0.27	0.25	-									
10. MSPSS_cg_	16	70.38	9.21	0.22	0.63**	0.39	0.06	−0.28	−0.08	0.11	0.41	0.32	-								
11. PCS_p_	16	53.23	3.73	0.23	−0.18	−0.08	0.05	0.43	−0.12	−0.10	0.26	−0.13	−0.07	-							
12. PCS_cg_	16	53.11	6.88	0.06	−0.10	−0.26	−0.15	−0.37	−0.42	0.32	0.05	0.01	−0.09	−0.25	-						
13. MCS_p_	16	46.37	12.96	0.60*	−0.17	−0.01	0.40	0.74**	0.00	−0.31	0.43	0.37	0.11	0.28	−0.29	-					
14. MCS_cg_	16	49.07	9.65	0.12	0.07	0.27	−0.12	0.03	0.38	0.02	0.20	0.14	0.56*	0.29	−0.54*	0.18	-				
15. SDM_p_	16	35.06	9.48	−0.14	−0.09	−0.25	−0.06	0.19	0.19	−0.09	−0.46	0.10	0.00	−0.10	−0.24	0.12	0.37	-			
16. SDM_cg_	14	32.21	13.31	−0.28	0.23	−0.28	0.08	0.20	0.00	0.12	−0.01	−0.05	0.56*	0.33	−0.45	0.20	0.70**	0.65*	-		
17. CARE_p_	16	40.31	9.80	−0.35	0.02	0.36	−0.20	−0.22	0.59*	−0.19	−0.08	0.02	0.22	0.02	−0.39	−0.38	0.56*	0.06	0.22	-	
18. CARE_cg_	14	46.79	12.22	0.19	0.42	−0.13	−0.04	−0.15	−0.32	0.14	0.38	−0.16	0.11	−0.04	0.21	−0.17	−0.14	−0.54*	−0.37	0.20	-

*p ≤ 0.05; **p ≤ 0.01. p, patient; cg, caregiver.

## Discussion

Considering cancer as a family disease, exploring patients and caregivers’ reaction to a cancer diagnosis and their alignment toward DM is becoming increasingly important, as it is the first step to create appropriate guidelines for healthcare providers and structure dyadic psychological support intervention for patients and caregivers’ dyads. Recent evidence showed that an alignment between patients’ and caregivers’ preference in DM may enhance the process of care for all the parties (31). In this pilot study, we investigated the psychological impact of a newly diagnosed PC on patients’ and caregivers’ dyads and their alignment in the DM. Some questions which guided our investigation were the following: Does the dyad really agree on how to be involved in the DM? How patients’ and caregivers’ dyads react and cope to a cancer diagnosis? How the psychological reaction of patients’ influences the one of the caregivers’ and vice versa?

In our sample, participants were predominantly middle-aged adults, married and well-educated. The mean age of the PC patients is similar as the one reported in Europe (2). Specifically, almost all the dyads were spouses, as reported in our previous systematic review (31).

Our study showed that only a quarter of the dyad agreed in sharing the DM responsibility, although taken separately more patients and caregivers reported preferring this modality. This finding is in contrast with our first hypothesis, that was a high agreement among the dyads. This result suggested that patients and caregivers did not always share the preference on how to be involved in decision making, and this could lead to friction within the dyad, even if not explicit, and worsen the psychological well-being of both. However, this result was in line with our systematic review, except for the agreement in the DM, because we found that half of the patients and caregivers preferred to share decision-making responsibility within the dyads, or that the patients have an active role in decision-making, after taking the caregiver’s opinion into account (31). From a clinical standpoint, it was interesting that no sons preferred to share the responsibility of the decision with the patients. This finding suggests the diversity of the relationship between parents and children and between couples. It would be very interesting to investigate more what factors lead to this difference. Moreover, dyads reported experiencing an open and flowing exchange of ideas between them and in this variable, more than half of the dyad agreed on their communication style, as hypothesized in our second hypothesis. A greater communication style was obtained with a good social support in the patients’ sample. This result suggests that patients in our sample talked openly to the caregivers, communicating their needs, and feeling supported. However, for the caregiver, this meant having to accommodate more of the patient’s fears, suffering and frailty, raising their own anxiety levels. This has been frequently demonstrated in the literature (73–76) and lead to the need of specific psychological intervention for caregivers, consistent with the literature (77).

As reported in literature, patients and caregivers experienced high level of psychological distress (e.g., anxiety and depression) after having received a cancer diagnosis (6, 31, 78–81). This finding is in line with our third hypothesis. However, in our sample patients had higher psychological distress than caregivers, and this is not in line with literature (1, 10). Patients and caregivers may exhibit asynchrony in their emotional state, e.g., caregivers may not be fully aware of the significance of the diagnosis and what it will entail, while patients may experience more fears about the uncertainty of the future, related to fear of death or recurrence (6). However, our sample reported a mean score in the QoL above the cut off, showing a good QoL. In particular, older patients had a greater QoL.

Regarding the mutual influence of patients and caregivers, our results showed correlations between patients’ and caregiver’ different psychological variables. It is interesting to notice that there was a correlation between patients’ and caregiver’ perceived involved in the shared decision-making. This means that more patients’ felt themselves involved in the decision-making, more the caregivers felt the same. This result supported the importance of involving even the caregivers in the SDM and of training medical team in how to speak with dyads. In addition, the perception of patients as highly involved in DM by medical team leads caregivers to perceive medical team as more empathetic. This result was in line with the conceptual framework proposed by Laidsaar-Powell et al. (20), according to which caregivers are involved in various ways in the decision-making process, transforming the classic patient-physician interaction into a triadic relationship (patient, caregiver, and medical team) (19, 35, 82).

Another important finding was the negative correlation between patients’ level of anxiety and caregivers’ level of depression. Patients’ perceived anxiety about the future would be poorly managed by caregivers with high levels of depression, who would exhibit low levels of activation and planning. Moreover, our results suggested that caregivers’ anxiety was related to patients’ empathy toward medical team, and this might suggest that patients need someone they can trust and who is able to accommodate their needs and frustrations.

Another interesting aspect emerging from our results was the greater empathy and involvement in the DM with medical team. This means that health professionals have taken charge of the patient and caregiver, providing the appropriate information in a clear manner. They made the dyads feel comfortable, inspiring confidence in them. It is important to underline that patient with high level of anxiety reported higher level of medical team’ empathy and communications. This might suggest that health professionals have been able to accommodate patients’ anxiety and that at the same time patients with a lot of anxiety need medical team who are more empathetic and available for dialog.

Before concluding, it is important to point out some limitations of this pilot study. As the sample is very small, this pilot study cannot guarantee the magnitude of the response rate in the main survey. Questionnaires to assess psychological variables are self-report and this may lead potential bias. The majority of the sample is composed by couples, and this may limit the generalization of results for other type of dyads. From a clinical point of view, it would have been interesting to have a sample size such that we could infer differences depending on the relationship between patient and caregiver. A son compared to his wife feels differently involved and less entitled to make decisions. Moreover, we didn’t collect information about the relationship’s quality.

Despite lack of statistical power and these limitations, results of this study are promising and important to consider when designing future research in this area.

## Conclusion

Our results suggest the importance of involving both patients and caregivers in decision making. It is necessary better investigate how they want to be involved in order to have a good degree of agreement in the dyad. Cancer certainly is a family disease that impacts both members of the dyad, and it is necessary to structure specific interventions for both individuals and dyads, taking into account the mutual influence they have when facing a cancer diagnosis.

Finally, this pilot study provided us with the necessary information to move forward with the longitudinal study. However, because of the large time difference between prostate cancer and breast cancer, in terms of waiting time between diagnosis and treatment initiation, and because of the difficulty in enrolling patients with prostate cancer, we decided to focus only on dyads with breast cancer.

## Data availability statement

The raw data supporting the conclusions of this article will be made available by the authors, without undue reservation.

## Ethics statement

The studies involving humans were approved by Institutional Review Board (or Ethics Committee) of the European Institute of Oncology, IRCCS. The studies were conducted in accordance with the local legislation and institutional requirements. The participants provided their written informed consent to participate in this study.

## Author contributions

CC: Conceptualization, Formal analysis, Investigation, Methodology, Writing – original draft, Writing – review & editing. SP: Methodology, Writing – review & editing. SO: Conceptualization, Supervision, Writing – review & editing. PG: Formal analysis, Writing – review & editing. GP: Supervision, Writing – review & editing.

## References

[1] Varner S Lloyd G Ranby KW Callan S Robertson C Lipkus IM. Illness uncertainty, partner support, and quality of life: A dyadic longitudinal investigation of couples facing prostate cancer. Psycho-Oncology. (2019) 28:2188–94. doi: 10.1002/pon.5205, PMID: 31418505

[2] Yiannopoulou KG Anastasiou AI Kontoangelos K Papageorgiou C Anastasiou IP. Cognitive and Psychological Impacts of Different Treatment Options for Prostate Cancer: A Critical Analysis. Current Urol. (2020) 14:169–77. doi: 10.1159/000499242, PMID: 33488334 PMC7810213

[3] Maggi M Gentilucci A Salciccia S Gatto A Gentile V Colarieti A. Psychological impact of different primary treatments for prostate cancer: A critical analysis. Andrologia. (2019) 51:e13157. doi: 10.1111/and.13157 30281167

[4] Tidd-Johnson A Sebastian SA Co EL Afaq M Kochhar H Sheikh M. Prostate cancer screening: Continued controversies and novel biomarker advancements. Current urol. (2022) 16:197–206. doi: 10.1097/CU9.0000000000000145, PMID: 36714234 PMC9875204

[5] Scherr K Delaney RK Ubel P Kahn VC Hamstra D Wei JT. Preparing Patients with Early Stage Prostate Cancer to Participate in Clinical Appointments Using a Shared Decision Making Training Video. Med Decis Mak. (2022) 42:364–74. doi: 10.1177/0272989X211028563, PMID: 34617827 PMC8918874

[6] Cincidda C Oliveri S Sanchini V Pravettoni G. The role of caregivers in the clinical pathway of patients newly diagnosed with breast and prostate cancer: A study protocol. Front Psychol. (2022) 13, 962634. doi: 10.3389/fpsyg.2022.962634, PMID: 36405193 PMC9667064

[7] Cincidda C Pizzoli SFM Pravettoni G. Remote Psychological Interventions for Fear of Cancer Recurrence: Scoping Review. JMIR Cancer. (2022) 8:e29745. doi: 10.2196/29745, PMID: 35014956 PMC8790693

[8] Ilie G Rendon R Mason R MacDonald C Kucharczyk MJ Patil N. A Comprehensive 6-mo Prostate Cancer Patient Empowerment Program Decreases Psychological Distress Among Men Undergoing Curative Prostate Cancer Treatment: A Randomized Clinical Trial. Eur Urol. (2023) 83:561–70. doi: 10.1016/j.eururo.2023.02.009, PMID: 36822969

[9] Muzzatti B Bomben F Flaiban C Piccinin M Annunziata MA. Quality of life and psychological distress during cancer: a prospective observational study involving young breast cancer female patients. BMC Cancer. (2020) 20:758. doi: 10.1186/s12885-020-07272-8, PMID: 32791959 PMC7425052

[10] Masiero M Busacchio D Guiddi P Arnaboldi P Musi G De Cobelli O. Quality of life and psycho-emotional wellbeing in bladder cancer patients and their caregivers: a comparative analysis between urostomy versus ileal orthotopic neobladder. E Cancer Med Sci. (2021) 15:1163. doi: 10.3332/ecancer.2021.1163, PMID: 33680077 PMC7929779

[11] Ruane-McAteer E Porter S O’Sullivan JM Santin O Prue G. Active surveillance for favorable-risk prostate cancer: Is there a greater psychological impact than previously thought? A systematic, mixed studies literature review. Psycho-Oncology. (2017) 26:1411–21. doi: 10.1002/pon.4311, PMID: 27862602

[12] Pravettoni G. Cutica I. Righetti S. Mazzocco K. (2016). Decisions and involvement of cancer patient survivors: a moral imperative. Journal of Healthcare Leadership, Volume 8, 121–125. doi: 10.2147/JHL.S115434 29355188 PMC5741003

[13] Arnaboldi P. Oliveri S. Vergani L. Marton G. Guiddi P. Busacchio D.. (2020). The clinical-care focused psychological interview (CLiC): a structured tool for the assessment of cancer patients’ needs. Ecancermedicalscience, 14. doi: 10.3332/ecancer.2020.1000 PMC7032941 32153655

[14] Lamore K Montalescot L Untas A. Treatment decision-making in chronic diseases: What are the family members’ roles, needs and attitudes? A systematic review. Patient Educ Couns. (2017) 100:2172–81. doi: 10.1016/j.pec.2017.08.003, PMID: 28838630

[15] Russo G. A. Oliveri S. Cincidda C. Guiddi P. Pravettoni G. (2020). Exploring Public Attitude toward Biofeedback Technologies: Knowledge, Preferences and Personality Tendencies. Journal of Public Health Research, 9(4), doi: 10.4081/jphr.2020.1782 PMC7662746 33209858

[16] Marton G. Pizzoli S. F. M. Vergani L. Mazzocco K. Monzani D. Bailo L.. (2021). Patients’ health locus of control and preferences about the role that they want to play in the medical decision-making process. Psychology, Health \u0026amp; Medicine, 26, 260–266. doi: 10.1080/13548506.2020.1748211 32323553

[17] Covvey J. R. Kamal K. M. Gorse E. E. Mehta Z. Dhumal T. Heidari E.. (2019). Barriers and facilitators to shared decision-making in oncology: a systematic review of the literature. Supportive Care in Cancer, 27, 1613–1637. doi: 10.1007/s00520-019-04675-7 30737578

[18] Malhotra C. Kanesvaran R. Barr Kumarakulasinghe N. Tan S. Xiang L. Tulsky J. A.. (2020). Oncologist‐patient‐caregiver decision‐making discussions in the context of advanced cancer in an Asian setting. Health Expectations, 23, 220–228. doi: 10.1111/hex.12994 31682064 PMC6978867

[19] Renzi C Riva S Masiero M Pravettoni G. The choice dilemma in chronic hematological conditions: Why choosing is not only a medical issue? A psycho-cognitive perspective. Crit Rev Oncol Hematol. (2016) 99:134–40. doi: 10.1016/j.critrevonc.2015.12.010, PMID: 26762858

[20] Laidsaar-Powell R Butow P Charles C Gafni A Entwistle V Epstein R. The TRIO Framework: Conceptual insights into family caregiver involvement and influence throughout cancer treatment decision-making. Patient Educ Couns. (2017) 100:2035–46. doi: 10.1016/j.pec.2017.05.014, PMID: 28552193

[21] Oliveri S Pravettoni G Fioretti C Hansson MG. Let the individuals directly concerned decide: a solution to tragic choices in genetic risk information. Public Health Genomics. (2016) 19:307–13. doi: 10.1159/000448913, PMID: 27603671

[22] Del Piccolo L. Goss C. Bottacini A. Rigoni V. Mazzi M. A. Deledda G. (2014). Asking questions during breast cancer consultations: Does being alone or being accompanied make a difference? European Journal of Oncology Nursing, 18, 299– 304. doi: 10.1016/j.ejon.2014.02.001 24629501

[23] Gómez-Vírseda C. de Maeseneer Y. Gastmans C. (2020). Relational autonomy in end-of-life care ethics: a contextualized approach to real-life complexities. BMC Medical Ethics, 21, 50. doi: 10.1186/s12910-020-00495-1 32605569 PMC7325052

[24] Rapley T. Distributed decision making: the anatomy of decisions-in-action. Sociol Health Illn. (2008) 30:429–44. doi: 10.1111/j.1467-9566.2007.01064.x, PMID: 18194358

[25] Epstein RM Street RL. Shared mind: communication, decision making, and autonomy in serious illness. Ann Family Med. (2011) 9:454–61. doi: 10.1370/afm.1301, PMID: 21911765 PMC3185482

[26] Elwyn G Lloyd A May C van der Weijden T Stiggelbout A Edwards A. Collaborative deliberation: a model for patient care. Patient Educ Couns. (2014) 97:158–64. doi: 10.1016/j.pec.2014.07.027, PMID: 25175366

[27] Légaré F Stacey D Gagnon S Dunn S Pluye P Frosch D. Validating a conceptual model for an inter-professional approach to shared decision making: a mixed methods study. J Eval Clin Pract. (2011) 17:554–64. doi: 10.1111/j.1365-2753.2010.01515.x, PMID: 20695950 PMC3170704

[28] Grignoli N. Di Bernardo V. Malacrida R. (2018). New perspectives on substituted relational autonomy for shared decision-making in critical care. Critical Care, 22, 260. doi: 10.1186/s13054-018-2187-6 30309384 PMC6182794

[29] Acquati C Kayser K. Dyadic Coping Across the Lifespan: A Comparison Between Younger and Middle-Aged Couples With Breast Cancer. Front Psychol. (2019) 10, 404. doi: 10.3389/fpsyg.2019.00404, PMID: 30941068 PMC6433932

[30] Chen M Gong J Cao Q Luo X Li J Li Q. A literature review of the relationship between dyadic coping and dyadic outcomes in cancer couples. Eur J Oncol Nurs. (2021) 54:102035. doi: 10.1016/j.ejon.2021.102035, PMID: 34520996

[31] Cincidda C Pizzoli SFM Ongaro G Oliveri S Pravettoni G. Caregiving and Shared Decision Making in Breast and Prostate Cancer Patients: A Systematic Review. Curr Oncol. (2023) 30:803–23. doi: 10.3390/curroncol30010061, PMID: 36661710 PMC9857468

[32] Schumm K Skea Z McKee L N’Dow J. ‘They’re doing surgery on two people’: a meta- ethnography of the influences on couples’ treatment decision making for prostate cancer. Health Expect. (2010) 13:335–49. doi: 10.1111/j.1369-7625.2010.00624.x, PMID: 20860778 PMC5060544

[33] Dionne-Odom JN Ejem D Wells R Barnato AE Taylor RA Rocque GB. How family caregivers of persons with advanced cancer assist with upstream healthcare decision-making: A qualitative study. PLoS One. (2019) 14:e0212967. doi: 10.1371/journal.pone.0212967, PMID: 30865681 PMC6415885

[34] Trevino KM Prigerson HG Shen MJ Tancredi DJ Xing G Hoerger M. Association between advanced cancer patient-caregiver agreement regarding prognosis and hospice enrollment. Cancer. (2019) 125:3259–65. doi: 10.1002/cncr.32188, PMID: 31145833 PMC6717015

[35] Tulsky JA Steinhauser KE LeBlanc TW Bloom N Lyna PR Riley J. Triadic agreement about advanced cancer treatment decisions: Perceptions among patients, families, and oncologists. Patient Educ Couns. (2022) 105:982–6. doi: 10.1016/j.pec.2021.08.001, PMID: 34384640

[36] Ullrich A Theochari M Bergelt C Marx G Woellert K Bokemeyer C. Ethical challenges in family caregivers of patients with advanced cancer – a qualitative study. BMC Palliat Care. (2020) 19:70. doi: 10.1186/s12904-020-00573-6, PMID: 32423444 PMC7236546

[37] Blackler L. Compromised Autonomy. J Hosp Palliat Nurs. (2016) 18:184–91. doi: 10.1097/NJH.0000000000000264

[38] Hobbs GS Landrum MB Arora NK Ganz PA van Ryn M Weeks JC. The role of families in decisions regarding cancer treatments. Cancer. (2015) 121:1079–87. doi: 10.1002/cncr.29064, PMID: 25708952 PMC4368490

[39] Laryionava K Hauke D Heußner P Hiddemann W Winkler EC. “Often Relatives are the Key […]” –Family Involvement in Treatment Decision Making in Patients with Advanced Cancer Near the End of Life. Oncologist. (2021) 26:e831–7. doi: 10.1002/onco.13557, PMID: 33037846 PMC8100569

[40] Jones RA Steeves R Ropka ME Hollen P. Capturing treatment decision making among patients with solid tumors and their caregivers. Oncol Nurs Forum. (2013) 40:E24–31. doi: 10.1188/13.ONF.E24-E31, PMID: 23269778 PMC3634347

[41] Sinfield P Baker R Camosso-Stefinovic J Colman AM Tarrant C Mellon JK. Men’s and carers’ experiences of care for prostate cancer: a narrative literature review. Health Expect. (2009) 12:301–12. doi: 10.1111/j.1369-7625.2009.00546.x, PMID: 19754693 PMC5060497

[42] Schumacher FA Helenowski IB Oswald LB Gonzalez BD Benning JT Morgans AK. Treatment decision-making in metastatic prostate cancer: perceptions of locus of control among patient, caregiver, and physician triads. Patient Prefer Adherence. (2022) 16:235–44. doi: 10.2147/PPA.S334827, PMID: 35125865 PMC8811793

[43] Siminoff LA Wilson-Genderson M Barta S Thomson MD. Hematological cancer patient-caregiver dyadic communication: A longitudinal examination of cancer communication concordance. Psycho-Oncology. (2020) 29:1571–8. doi: 10.1002/pon.5458, PMID: 32627258 PMC8474783

[44] DuBenske LL Chih M-Y Gustafson DH Dinauer S Cleary JF. Caregivers’ participation in the oncology clinic visit mediates the relationship between their information competence and their need fulfillment and clinic visit satisfaction. Patient Educ Couns. (2010) 81:S94–9. doi: 10.1016/j.pec.2010.08.022, PMID: 20880656 PMC2993845

[45] Joosten EAG DeFuentes-Merillas L de Weert GH Sensky T van der Staak CPF de Jong CJ. Systematic Review of the Effects of Shared Decision-Making on Patient Satisfaction, Treatment Adherence and Health Status. Psychother Psychosom. (2008) 77:219–26. doi: 10.1159/000126073, PMID: 18418028

[46] Ozdemir S Ng S Chaudhry I Teo I Malhotra C Finkelstein EA. Caregiver-Reported Roles in Treatment Decision Making in Advanced Cancer and Associated Caregiving Burden and Psychological Distress: A Longitudinal Study. Med Decis Mak. (2023) 43:191–202. doi: 10.1177/0272989X221125408 36113405

[47] Gray RE Fitch MI Phillips C Labrecque M Klotz L. Presurgery Experiences of Prostate Cancer Patients and Their Spouses. Cancer Pract. (1999) 7:130–5. doi: 10.1046/j.1523-5394.1999.07308.x, PMID: 10352075

[48] Lafaye A Petit S Richaud P Houédé N Baguet F Cousson-Gélie F. Dyadic effects of coping strategies on emotional state and quality of life in prostate cancer patients and their spouses. Psycho-Oncology. (2014) 23:797–803. doi: 10.1002/pon.3483, PMID: 24493581

[49] Giordano A Mattarozzi K Pucci E Leone M Casini F Collimedaglia L. Participation in medical decision-making: Attitudes of Italians with multiple sclerosis. J Neurol Sci. (2008) 275:86–91. doi: 10.1016/j.jns.2008.07.026, PMID: 18786682

[50] Shin DW Cho J Kim SY Yang HK Park K Kweon S-S. Patients’ and family caregivers’ understanding of the cancer stage, treatment goal, and chance of cure: A study with patient-caregiver-physician triad. Psycho-Oncology. (2018) 27:106–13. doi: 10.1002/pon.4467, PMID: 28603876

[51] Shin DW Cho J Roter DL Kim SY Sohn SK Yoon M-S. Preferences for and experiences of family involvement in cancer treatment decision-making: patient-caregiver dyads study. Psycho-Oncology. (2013) 22:2624–31. doi: 10.1002/pon.3339 23893390

[52] Shin DW Cho J Roter DL Kim SY Yang HK Park K. Attitudes Toward Family Involvement in Cancer Treatment Decision Making: The Perspectives of Patients, Family Caregivers, and Their Oncologists. Psycho-Oncology. (2017) 26:770–8. doi: 10.1002/pon.4226, PMID: 27437905

[53] Degner LF Sloan JA Venkatesh P. The Control Preferences Scale. Canadian J Nurs Res = Revue Canadienne de Recherche En Sciences Infirmieres. (1997) 29:21–43. PMID: 9505581

[54] Schaefer MT Olson DH. Assessing Intimacy: The Pair Inventory*. J Marital Fam Ther. (1981) 7:47–60. doi: 10.1111/j.1752-0606.1981.tb01351.x

[55] Constant E Vallet F Nandrino J-L Christophe V. Personal assessment of intimacy in relationships: Validity and measurement invariance across gender. Eur Rev Appl Psychol. (2016) 66:109–16. doi: 10.1016/j.erap.2016.04.008

[56] Miller SM. Monitoring and blunting: Validation of a questionnaire to assess styles of information seeking under threat. J Pers Soc Psychol. (1987) 52:345–53. doi: 10.1037/0022-3514.52.2.345, PMID: 3559895

[57] Rees CE Bath PA. The psychometric properties of the Miller Behavioural Style Scale with adult daughters of women with early breast cancer: a literature review and empirical study. J Adv Nurs. (2000) 32:366–74. doi: 10.1046/j.1365-2648.2000.01485.x, PMID: 10964184

[58] Zigmond AS Snaith RP. The Hospital Anxiety and Depression Scale. Acta Psychiatr Scand. (1983) 67:361–70. doi: 10.1111/j.1600-0447.1983.tb09716.x 6880820

[59] Dunbar M Ford G Hunt K Der G. A confirmatory factor analysis of the Hospital Anxiety and Depression scale: Comparing empirically and theoretically derived structures. Br J Clin Psychol. (2000) 39:79–94. doi: 10.1348/014466500163121, PMID: 10789030

[60] Annunziata MA Muzzatti B Altoè G. Defining Hospital Anxiety and Depression Scale (HADS) structure by confirmatory factor analysis: a contribution to validation for oncological settings. Ann Oncol. (2011) 22:2330–3. doi: 10.1093/annonc/mdq750 21339383

[61] Iani L Lauriola M Costantini M. A confirmatory bifactor analysis of the hospital anxiety and depression scale in an Italian community sample. Health Qual Life Outcomes. (2014) 12:84. doi: 10.1186/1477-7525-12-84, PMID: 24902622 PMC4054905

[62] Bell ML Fairclough DL Fiero MH Butow PN. Handling missing items in the Hospital Anxiety and Depression Scale (HADS): a simulation study. BMC Res Notes. (2016) 9:479. doi: 10.1186/s13104-016-2284-z, PMID: 27770833 PMC5075158

[63] Bjelland I Dahl AA Haug TT Neckelmann D. The validity of the Hospital Anxiety and Depression Scale. An updated literature review. J Psychosom Res. (2002) 52:69–77. doi: 10.1016/s0022-3999(01)00296-3 11832252

[64] Zimet GD Dahlem NW Zimet SG Farley GK. The Multidimensional Scale of Perceived Social Support. J Pers Assess. (1988) 52:30–41. doi: 10.1207/s15327752jpa5201_2

[65] Di Fabio A Palazzeschi L. Multidimensional Scale of Perceived Social Support (MSPSS): un contributo alla validazione italiana [Multidimensional Scale of Perceived Social Support (MSPSS): A contribution to Italian validation]. Counseling: Giornale Italiano Di Ricerca e Applicazioni. (2015) 8:127–40.

[66] Ware JE Jr Kosinski M Keller SD. A 12-Item Short-Form Health Survey. Med Care. (1996) 34:220–33. doi: 10.1097/00005650-199603000-00003 8628042

[67] Kodraliu G Mosconi P Groth N Carmosino G Perilli A Gianicolo EA. Subjective health status assessment: evaluation of the Italian version of the SF-12 Health Survey. Results from the MiOS Project J Epidemiol Biostat. (2001) 6:305–16. doi: 10.1080/135952201317080715, PMID: 11437095

[68] Kriston L Scholl I Hölzel L Simon D Loh A Härter M. The 9-item Shared Decision Making Questionnaire (SDM-Q-9). Development and psychometric properties in a primary care sample. Patient Educ Couns. (2010) 80:94–9. doi: 10.1016/j.pec.2009.09.034 19879711

[69] de Filippis R Aloi M Pilieci AM Boniello F Quirino D Steardo L. Psychometric Properties of the 9-Item Shared Decision-Making Questionnaire (SDM-Q-9): Validation of the Italian Version in a Large Psychiatric Clinical Sample. Clin Neuropsychiatry. (2022) 19:264–71. doi: 10.36131/cnfioritieditore20220408, PMID: 36101644 PMC9442857

[70] Mercer SW. The consultation and relational empathy (CARE) measure: development and preliminary validation and reliability of an empathy-based consultation process measure. Fam Pract. (2004) 21:699–705. doi: 10.1093/fampra/cmh621, PMID: 15528286

[71] Mercer SW Murphy DJ. Validity and reliability of the CARE Measure in secondary care. Clin Govern Int J. (2008) 13:269–83. doi: 10.1108/14777270810912969

[72] Natali F Corradini L Sconza C Taylor P Furlan R Mercer SW. Development of the Italian version of the Consultation and Relational Empathy (CARE) measure: translation, internal reliability, and construct validity in patients undergoing rehabilitation after total hip and knee arthroplasty. Disabil Rehabil. (2023) 45:703–8. doi: 10.1080/09638288.2022.2037742, PMID: 35191359

[73] Karimi Moghaddam Z Rostami M Zeraatchi A Mohammadi Bytamar J Saed O Zenozian S. Caregiving burden, depression, and anxiety among family caregivers of patients with cancer: An investigation of patient and caregiver factors. Front Psychol. (2023) 14:1059605 doi: 10.3389/fpsyg.2023.1059605, PMID: 37057172 PMC10086361

[74] Mishra S Gulia A Satapathy S Gogia A Sharma A Bhatnagar S. Caregiver burden and quality of life among family caregivers of cancer patients on chemotherapy: A prospective observational study. Indian J Palliat Care. (2021) 27:109. doi: 10.4103/IJPC.IJPC_180_20, PMID: 34035627 PMC8121224

[75] Park S Mazanec SR Burant CJ Bajor D Douglas SL. Caregiver Burden in Distance Caregivers of Patients with Cancer. Curr Oncol. (2022) 29:8967–74. doi: 10.3390/curroncol29110704, PMID: 36421357 PMC9689057

[76] Rohith K Patel A. The burden of cancer caregivers: Time to acknowledge and start caring for the carers. Cancer Res Stat Treatment. (2022) 5:309. doi: 10.4103/crst.crst_176_22

[77] Newell SA. Systematic Review of Psychological Therapies for Cancer Patients: Overview and Recommendations for Future Research. CancerSpectrum Knowledge Environ. (2002) 94:558–84. doi: 10.1093/jnci/94.8.558, PMID: 11959890

[78] Edwards B Clarke V. The psychological impact of a cancer diagnosis on families: The influence of family functioning and patients’ illness characteristics on depression and anxiety. Psycho-Oncology. (2004) 13:562–76. doi: 10.1002/pon.773, PMID: 15295777

[79] Hodges LJ Humphris GM Macfarlane G. A meta-analytic investigation of the relationship between the psychological distress of cancer patients and their carers. Soc Sci Med. (2005) 60:1–12. doi: 10.1016/j.socscimed.2004.04.018, PMID: 15482862

[80] Segrin C Badger TA. Psychological distress in different social network members of breast and prostate cancer survivors. Res Nurs Health. (2010) 33:450–64. doi: 10.1002/nur.20394 20672304 PMC2966868

[81] Stenberg U Ruland CM Miaskowski C. Review of the literature on the effects of caring for a patient with cancer. Psycho-Oncology. (2010) 19:1013–25. doi: 10.1002/pon.1670 20014159

[82] LeBlanc TW Bloom N Wolf SP Lowman SG Pollak KI Steinhauser KE. Triadic treatment decision-making in advanced cancer: a pilot study of the roles and perceptions of patients, caregivers, and oncologists. Support Care Cancer. (2018) 26:1197–205. doi: 10.1007/s00520-017-3942-y, PMID: 29101469

